# Insights into the Current and Possible Future Use of Opioid Antagonists in Relation to Opioid-Induced Constipation and Dysbiosis

**DOI:** 10.3390/molecules28237766

**Published:** 2023-11-24

**Authors:** Nariman Essmat, Dávid Árpád Karádi, Ferenc Zádor, Kornél Király, Susanna Fürst, Mahmoud Al-Khrasani

**Affiliations:** 1 Department of Pharmacology and Pharmacotherapy, Faculty of Medicine, Semmelweis University, Nagyvárad tér 4, H-1445 Budapest, Hungary; nariman.gomaa@phd.semmelweis.hu (N.E.); karadi.david_arpad@med.semmelweis-univ.hu (D.Á.K.); zador.ferenc@pharma.semmelweis-univ.hu (F.Z.); kiraly.kornel@med.semmelweis-univ.hu (K.K.); furst.zsuzsanna@med.semmelweis-univ.hu (S.F.); 2 Department of Pharmacology and Toxicology, Faculty of Pharmacy, Zagazig University, Zagazig 44519, Egypt

**Keywords:** OIC, opioid antagonists, PAMORAs, gut-selective MOR antagonists, OID

## Abstract

Opioid receptor agonists, particularly those that activate µ-opioid receptors (MORs), are essential analgesic agents for acute or chronic mild to severe pain treatment. However, their use has raised concerns including, among others, intestinal dysbiosis. In addition, growing data on constipation-evoked intestinal dysbiosis have been reported. Opioid-induced constipation (OIC) creates an obstacle to continuing treatment with opioid analgesics. When non-opioid therapies fail to overcome the OIC, opioid antagonists with peripheral, fast first-pass metabolism, and gastrointestinal localized effects remain the drug of choice for OIC, which are discussed here. At first glance, their use seems to only be restricted to constipation, however, recent data on OIC-related dysbiosis and its contribution to the appearance of several opioid side effects has garnered a great of attention from researchers. Peripheral MORs have also been considered as a future target for opioid analgesics with limited central side effects. The properties of MOR antagonists counteracting OIC, and with limited influence on central and possibly peripheral MOR-mediated antinociception, will be highlighted. A new concept is also proposed for developing gut-selective MOR antagonists to treat or restore OIC while keeping peripheral antinociception unaffected. The impact of opioid antagonists on OIC in relation to changes in the gut microbiome is included.

## 1. Introduction

The research history of the opioid structure is traced back to the 19th century when morphine was isolated by Friedrich Wilhelm Sertürner [1]. Since then, morphine has been considered one of the most important analgesic agents used to manage acute and chronic mild to severe pain. Next, besides the great benefit of opioid analgesics in the management of pain, opioid abuse liability-related effects, respiratory depression, apnea, and death have opened avenues for developing agents to overcome opioid overdose and opioid use disorder. Furthermore, in addition to constipation, the accumulation of large amounts of data on the involvement of peripheral opioid receptors in the development of opioid analgesic tolerance, alterations in intestinal bacterial composition, and their consequences have opened new avenues for repurposing medications including opioid antagonists.

Opioid agonists exert their antinociceptive actions through the activation of opioid receptors, particularly µ-opioid receptors (MORs), both peripherally and centrally (spinal and supraspinal MOR activation) [2]. Although the site of action of clinically available analgesics is considered to be central, MOR-mediated peripheral analgesia has also been identified in human and experimental pain models [3,4,5,6,7,8,9,10]. The current consensus is that opioid agonists are the most effective analgesics for treating mild to severe acute and chronic pain types, yet in the case of neuropathic pain, their effect is up for debate [11,12,13]. The peripheral distribution of MORs is not limited to the peripheral sensory fiber terminals that mediate peripheral antinociception, and they are also found in other tissues including the gastrointestinal tract where they can mediate constipation [14,15,16]. Activation of gastrointestinal MORs has advantageous therapy properties related to the treatment of diarrhea. However, MOR activation has also been identified as being implicated in the development of gastrointestinal dysbiosis, an imbalance in microbiome composition [17,18,19]. Recent data have indicated that opioid-induced dysbiosis (OID) is associated with the development of opioid analgesic tolerance, opioid-induced hyperalgesia, and the progression of chronic pain conditions such as neuropathic pain [4,20,21,22]. More recently, the notion of the role of peripherally acting opioid antagonists in improving the antinociceptive effects of opioid agonists by reversing opioid analgesic-induced alterations in the gastrointestinal microbiome has been proposed. Indeed, proposed mechanisms of OID are varied, including opioid-induced constipation (OIC) (for review see Zádori et al., 2022 [23]). This reflects the fact that, despite the greater use of opioid agonists for the treatment of pain, opioid antagonists have also gained relevant practical use in the clinical world such as in the present context of OID and OIC, among others, in relation to opioid overdose, opioid and/or alcohol maintenance treatment, and obesity. This review will pay attention to the pharmacology of opioid antagonists, including peripherally acting opioid antagonists, and their benefits for animals and human subjects complaining of OIC as a result of the activation of gastrointestinal MORs and its consequences, as covered in detail in Section 2. In addition, it will shed light on the hallmarks of relationships between antagonist treatment and the composition of the gastrointestinal microbiome as seen in OIC. This review will also discuss the association between direct and indirect anti-opioids and the gut microbiome under the condition of opioid treatment. Furthermore, the review aims to find an alternative and complementary avenue that identifies and develops peripherally selective opioid antagonists that overcome the adverse gastrointestinal effects of MOR analgesics, particularly OIC, without impacting either the central opioid analgesia or peripheral analgesia, which is still the subject of current and future research. In this context, we present a scenario-based overview related to the concepts of developing opioid antagonists in order to overcome OIC and its consequences on the intestinal microbiome and pain. Finally, the impact of food-derived opioids and probiotics on gut dysbiosis, when simultaneously taken with these antagonists, are also discussed.

## 2. Opioid Receptors

Opioid receptors are G protein-coupled receptors (GPCRs) that mediate a wide variety of physiological and pharmacological effects upon the binding of endogenous or exogenous peptides and non-peptide opioid agonists. To date, three primary opioid receptor (OR) types can be distinguished, µ-opioid receptors (MORs), δ-opioid receptors (DORs), and κ-opioid receptors (KORs), as well as nociceptin receptors (NOP-R). They can be found in the central nervous system (CNS) and the periphery including the enteral nervous system [24,25,26]. ORs interact preferentially with Gi and Go α-subunits of G proteins that show sensitivity for the pertussis toxin [27,28]. Upon the binding of agonists to ORs, the trimer structure of G-proteins dissociates to Gα and the Gβγ, which are involved in the regulation of several intracellular effectors such as enzymes, and ion channels [29,30]. The inhibition of adenylyl cyclase, activation of inwardly rectifying K^+^ channels, and inhibition of voltage activated Ca^2+^ channels occurred as a consequence of OR activation [31].

MORs are the primary target for the current opioid analgesic agents used in the management of pain. The major drawbacks of these agents, such as morphine, include the development of analgesic tolerance, addiction liability, and constipation [32]. It is worth noting that there is no tolerance for OIC [4], which is considered to be a peripheral gastrointestinal MOR-mediated effect, yet some research works have reported on the involvement of CNS MORs as well [33,34,35]. 

Neuroanatomical studies have localized all three opioid receptor types in the myenteric and submucosal plexuses of the enteral nervous system on muscular and secretory motor neurons and interneurons [16]. Activation of opioid receptors in the enteral nervous system inhibits the release of neurotransmitters from excitatory motor neurons and stimulates neurotransmitter release from inhibitory motor neurons, resulting in non-propulsive motility. Administration of exogenous opioids (e.g., morphine) for analgesic purposes does activate the peripheral MORs in the gastrointestinal tract and can evoke several adverse gastrointestinal effects, such as alteration in fluid dynamics, inhibition of gastric emptying, and intestinal coordinated propulsive activity, and can thereby increase transit time, all of which may contribute to postoperative ileus and OIC [16,23,36]. In fact, opioid agonist-induced slow gastric emptying, decreases in both small and large bowel transits as well as an increase in anal sphincter tone are together involved in the development of OIC [4,37,38,39,40]. Constipation, intestinal spasms, and abdominal pain are some of the clinical manifestations of the disproportions that occur between the small and large intestines [41]. Additionally, less pancreaticobiliary secretion and gut absorption lead to a harder, drier stool when MORs in the enteric nervous system are directly activated, they are also associated with a reduction in vasoactive intestinal peptide release. In contrast to clinically used opioid analgesics, such as morphine, oxycodone, and fentanyl, G protein-biased MOR agonists have been reported to have reduced adverse effects including constipation, but there is no scientific consensus on this issue [42,43,44].

The concept on the presence of subtypes of MOR in the CNS has been proposed and published [45,46,47]. However, to the best of our knowledge this proposal has not been elucidated in gastrointestinal tract. Thus, the current consensus of the opioid research field is that there are no known differences in the sequences of the central and peripheral receptors that can be exploited for drug discovery purposes. 

## 3. Evidence on the Peripheral Pharmacology of Opioid Antagonists in Relation to Opioid-Induced Constipation

Opioid-induced constipation is experienced by ~41% of patients and constitutes one of the obstacles that limit continuing opioid analgesic therapies, particularly in long-term use [48,49]. For instance, 8 weeks of treatment with opioid analgesics can cause OIC in an average of 4% of patients [48]. In addition, both OIC and opioid-induced bowel dysfunction appear in up to 80% of patients who take opioids [50]. In fact, OIC causes pain that may discourage patients from taking opioid analgesics. Therefore, several non-opioid therapies (laxatives, prokinetics, chloride channel activators), and opioid rotation, have been approved for the treatment of OIC before starting opioid antagonist therapy.

**Figure 1:**
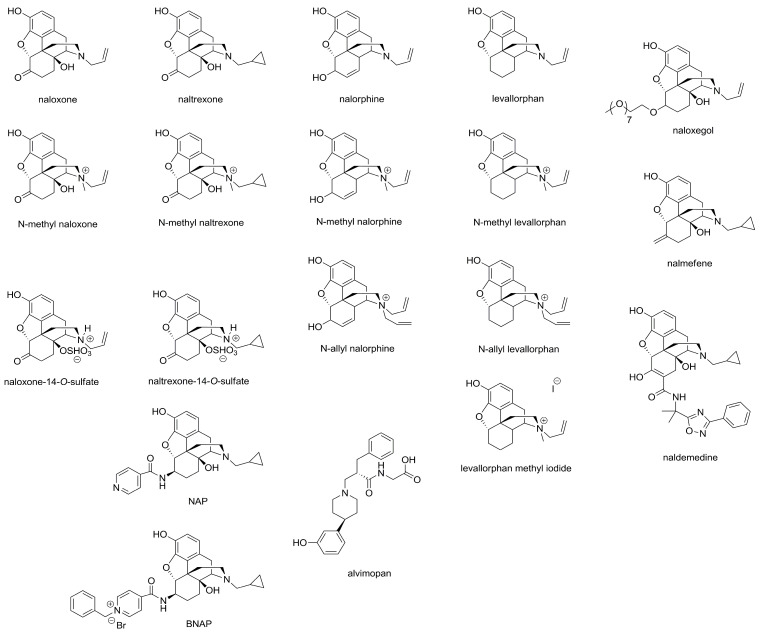
Chemical structure of the relevant opioid receptor antagonists.

To obtain the current medications that counteract the MOR-receptor-mediated side effects, great efforts have been exerted to develop opioid receptor antagonists such as naloxone, naltrexone, naloxegol, naldemedine, 6β-naltrexol, naltrexamine analogs (NAP and BNAP), and nalmefene, among others (Figure 1). The main purpose of developing opioid antagonists has been to inhibit opioid agonist-induced respiratory depression [51,52]. Aside from these effects, the pharmacological property of these antagonists creates a possibility of using them alone or in combination with opioid agonists or other medications to treat other clinical conditions such as obesity, disruptive impulse control, conduct disorder (kleptomania), opioid addiction, alcohol dependence, smoking [53,54,55], and OIC. With respect to opioid overdose, naloxone and nalmefene are being considered as rescuers. Unfortunately, when opioid antagonists are administered even in small doses they do reverse the well-controlled pain and make it agonizing, though controversial data have been reported relating to small doses of opioid antagonists and analgesia [56]. This section highlights the relevant steps in the development of opioid receptor antagonists, intended to reduce OIC (Figure 1) without notable impact on the analgesic action of the applied opioids.

### 3.1. Preclinical Studies 

Several preclinical studies have demonstrated the peripheral OR-mediated anti-opioid action of naloxone, naltrexone, and other morphinan-based quaternary opioid antagonists such N-methyl-naloxone or N-methyl-naltrexone (MNTX), as well as *N*-allyl-nalorphine or N-methyl-nalorphine or levallorphan methyl iodide, by co-administering them with systemic morphine or another opioid agonists in animal models designed to assess the antinociception and constipation induced by opioids [57,58,59,60,61,62,63,64,65,66]. Tavani and coworkers provided data on the ability of *N*-allyl-nalorphine to counteract the morphine-induced intestinal transit delay in rats to a comparable extent [67]. Prior treatment with either N-methyl-nalorphine or N-methyl-levallorphan abolishes morphine-induced constipation but not antinociception [64]. In another work, Bianchi and coworkers [59] investigated the peripheral and central antinociceptive or constipating component of systemic morphine alone or in combination with *N*-allyl-nalorphine, *N*-methyl-nalorphine, *N*-methyl-naloxone, and *N*-methyl-naltrexone or naloxone in mice. In this work, the authors provided data on the ability of tested quaternary antagonists to restore morphine-induced constipation and partially reduce morphine’s antinociception in this animal pain model. In another study, the peripheral selectivity of some quaternary antagonists has been found to be species-dependent [61]. This observation was based on the fact that *N*-methyl-levallorphan showed greater peripheral selectivity in mice than in rats, even compared to *N*-allyl-levallorphan, *N*-methyl-nalorphine, and *N*-methyl-naloxone. Both *N*-methyl-levallorphan and *N*-methyl-nalorphine were the most potent in preventing constipation evoked by subcutaneous (s.c.) morphine in a charcoal meal assay. In a study carried out by Anselmi and coworkers, they showed that s.c. or intraperitoneally administered MNTX antagonized the chronic morphine treatment-induced total gastrointestinal transit delay, with no effect being measured in naïve guinea pigs’ gastrointestinal transit, indicating the sensitivity of OIC to MNTX [68].

Another strategy for developing peripheral MOR antagonists entails the synthesis of the 6β-*N*-heterocyclic-substituted naltrexamine derivative NAP, which has been reported to produce a measurable increase in the intestinal motility of morphine-treated mice [69,70]. In fact, some of these analogs, such as BNAP, showed an affinity for more than one opioid receptor subtype [71]. Kanemasa and co-workers reported that naldemedine, in certain doses, inhibits OIC without affecting analgesia or withdrawal in rats. The measured effect in this study was attributed to MORs, because the applied agonists were MOR-selective agonists, though naldemedine has displayed comparable affinity for MOR, DOR, and KOR in receptor binding assays [72]. Likewise, naloxegol, a pegylated derivative of naloxone, can antagonize the OIC in rat experiments where morphine was the test agonist [73]. Alvimopan is a fully synthetic zwitterionic piperidine derivative that is structurally completely different from other clinically used opioid antagonists. Based on affinity studies, alvimopan has been proven to be a MOR antagonist displaying an affinity five times higher for MOR and five times lower for both DOR and KOR than naloxone. The route of administration of alvimopan dictates its site-of-distribution profile within the peripheral tissue, namely peripheral tissues or the entire gastrointestinal tract when administered s.c. or orally, respectively [74,75]. Furthermore, zwitterionic opioid antagonists, namely naloxone-14-*O*-sulfate and naltrexone-14-*O*-sulfate have also been reported previously; however, their impact on OIC, to the best of our knowledge, has not yet been elucidated [76,77].

### 3.2. Human Studies

In humans, the real breakthrough was the introduction of methylnaltrexone into clinical practice to counteract the OIC in doses that are devoid of CNS effects [78,79,80].

A group of peripherally acting μ-opioid receptor antagonists (PAMORAs), known as methylnaltrexone, naldemedine, and naloxegol, have been approved to treat the OIC of patients with noncancer pain [81]. These agents selectively block peripheral MORs without affecting central opioid analgesia [82]. The first used PAMORA was MNTX, which is applied subcutaneously or orally; however, its oral availability is very low. as indicated by the large difference between the doses given orally and subcutaneously. Its first approved indication is to manage OIC in cancer and noncancer patients having a poor response to conventional laxatives in a palliative care setting [83].

In a randomized placebo-controlled trial, Michna et al. demonstrated that both daily and alternate-day administration of MNTX significantly improved rescue-free bowel movements compared to a placebo in patients receiving opioid therapy for noncancer pain. The study reported favorable number-needed-to treat values, namely 5 to 14 for MNTX compared to a placebo, indicating the therapeutic efficacy of MNTX [84]. Furthermore, recent research has explored the oral administration of MNTX in patients with chronic noncancer pain. In a published phase 3 study, Rauck et al. investigated three different doses (150, 300, and 450 mg) and found that the 450 mg dose was the most effective with 28.0% of administrations achieving rescue-free bowel movement within four hours of treatment, as opposed to 18.8% after placebo [85]. 

Naloxegol acts on peripheral opioid receptors to counteract OIC. Clinical trials have demonstrated the efficacy of oral naloxegol 25 mg administration in improving straining, stool consistency, and the frequency of fully spontaneous bowel movements compared to a placebo in noncancer pain patients [86]. Another FDA-approved PAMORA for OIC treatment is naldemedine. Clinical trials, namely COMPOSE-1 and COMPOSE-2, involving patients with persistent noncancer pain demonstrated that naldemedine significantly increased bowel movements above baseline compared to a placebo [87]. COMPOSE-3, a 52-week placebo-controlled study involving 1241 patients randomly assigned to receive either naldemedine or a placebo further confirmed the superior efficacy of naldemedine in increasing the frequency of spontaneous bowel movements [88]. In patients with cancer pain, the efficacy of naldemedine to attenuate OIC was confirmed by a two-week controlled study (COMPOSE-4) [89]. It is worth noting that, in addition to PAMORAs, a fixed-dose combination of oxycodone hydrochloride and extended-release naloxone hydrochloride has been approved to reduce the occurrence of OIC in chronic pain patients. The delayed release of naloxone allows it to act as a local antagonist on opioid receptors in the gastrointestinal tract while having minimal impact on analgesia owing to its first-pass effect once administered per os to human or animal subjects [90,91]. With respect to alvimopan, in clinical trials it was proven to effectively inhibits constipation evoked by either loperamide [92] or chronic opioid treatment without affecting analgesia [93]. It has also been shown to prevent chronic opioid treatment-induced gastrointestinal side effects [94,95].

The aforementioned preclinical and clinical studies support the effectiveness of the peripherally acting opioid antagonists as well as those with systemic metabolism in counteracting OIC (Table 1). In addition, they have an advantage over the readily CNS-penetrating opioid antagonists with the negligible first-pass effect of being devoid of the reduction in opioid analgesia or reduced opioid antagonist-precipitated withdrawal.

Recent evidence of the deleterious effect of OIC on changes to the gastrointestinal microbiome has attracted many opioid researchers and clinicians [23,96]. Data presented in Section 4 answer the issue of whether these opioid antagonists could normalize the changes in the microbiome.

### 3.3. Safety of Current Opioid Antagonists for OIC Therapy

Long-term studies have been carried out to evaluate the safety of PAMORAs for OIC therapy. In a multicenter phase III study with 1034 chronic noncancer pain patients experiencing OIC, the administration of a daily 12 mg subcutaneous MNTX for 48 weeks resulted in significant improvements in various bowel-related parameters. Adverse events were mainly mild to moderate, and 15.2% of patients discontinued the study due to such events [97]. In addition, treatment with oral MNTX (150, 300, or 450 mg, or placebo once per day for 4 weeks, followed by as-needed use for 8 weeks) for OIC in patients with chronic noncancer pain showed a long-term safety profile comparable with a placebo, with no evidence of cardiac toxicity or opioid withdrawal [98]. Another long-term study intended to examine the tolerability of naloxegol (52 weeks) showed similar results with no new tolerability issues, and all adverse events reported were related to gastrointestinal transit [99]. The long-term safety and tolerability of naloxegol was also confirmed in a 52-week randomized placebo-controlled study [100]. With respect to naldemedine, in the COMPOSE-1 and COMPOSE-2 studies, patients were randomly assigned to receive either oral naldemedine 0.2 mg or a placebo daily for 12 weeks. Naldemedine was more effective in treating OIC in chronic noncancer pain patients. However, it had a higher rate of adverse events (15% vs. 7% in COMPOSE-1 and 16% vs. 7% in COMPOSE-2) compared to the placebo [87]. On the other hand, another study showed that naldemedine was well tolerated for 52 weeks and did not affect opioid analgesia or cause withdrawal symptoms. However, diarrhea was reported more frequently with naldemedine (11.0%) vs. placebo (5.3%) [101]. In a study designed to assess the safety of naloxone once combined with oxycodone, a 52-week treatment with oxycodone/naloxone found it be safe, well-tolerated, and typical of opioid treatment [102]. Alvimopan is only approved for short-term usage in hospitals for treatment of postoperative ileus due to the risk of myocardial infarction observed in several clinical studies [103,104].

The safety profile of all PAMORAs is comparable to most common symptoms, such as abdominal pain, diarrhea, and nausea/vomiting. However, the situation is different regarding the cardiac risks associated with alvimopan, as mentioned above. Moreover, it is essential to consider potential interactions between naloxegol and drugs that share the CP450 pathway [105]. It is also important to assess the use of naloxegol [106] and MNTX [107,108] in individuals with renal failure.

**Table 1:** MOR antagonist-mediated effects to reduce OIC that are based on a co-formulation product or a separate drug product.

Co-Formulated Product	Route of Administration	Purpose of the Combination	Reference
NX + oxycodone	per os	OIC	[109]
NX + oxycodone (1:2)	per os (prolonged release)	OIC	[90]
NX + oxycodone (1:2)	per os (prolonged release)	OIC	[91]
MNTX + opioid analgesics	s.c. + per os	OIC	[110]
MNTX + morphine, oxycodone, or fentanyl	s.c.(MTNX) (per os) morphine (continuous-release patches) fentanyl, oxycodone	OIC	[111]
MNTX + morphine	s.c.(MTNX) per os (morphine)	OIC	[84]
MNTX + morphine	per os	OIC	[85]
MNTX + morphine	s.c.(MTNX)	OIC	[112]
MNTX + morphine	s.c.(MTNX)	OIC	[113]
MNTX + morphine	s.c.(MTNX)	OIC	[114]
Naloxegol + morphine	per os	OIC	[86]
Naloxegol + morphine	per os	OIC	[100]
Naldemedine + morphine	per os	OIC	[87]
Naldemedine + opioid analgesic		OIC	[115]
Naldemedine + morphine	per os	OIC	[88]
Naldemedine + morphine	per os	OIC	[89]

## 4. Opioid Antagonists and the Gut Microbiome

A growing body of evidence has recently shed light on the critical role of the gastrointestinal microbiome, because changes in its composition can affect normal central and peripheral physiological functions in animals and humans, including nutrient absorption, immune status, and behavior [116,117]. With respect to pain, alterations in microbiome composition have been proven to be involved in pain pharmacology [118,119,120] and cognitive changes, which in turn are associated with chronic pain [121,122]. Nevertheless, it is important to note that the full picture on the degree to which the composition of the gut microbiome influences pain conditions, the bacterial lineages involved in these processes and their mechanisms are not yet fully understood. In fact, during treatment of chronic pain with opioid analgesics, the activation of MORs in the gastrointestinal tract and as a consequence OIC, which is one of the major participant factors in the development of gastrointestinal dysbiosis, should be considered prior to treatment with these types of medications. This implies creating a balance between the potential benefits and harms simultaneously or next to gastrointestinal MOR activation. Treatment with opioid analgesics results in OIC, which has largely participated in the peripheral adverse effect of opioids, yet no tolerance has been reported regarding OIC; therefore, strategies that are oriented to restoring the normal balance of microorganisms in the intestines seem to suit present clinical needs. With respect to OIC, the general consensus is that no opioid tolerance is related to constipation both in animal and human subjects even in long-term opioid treatment, as reviewed by Akbarali [123]. Indeed, there are animal data showing that chronic opioid exposure results in tolerance in the small intestine and the upper part of gastrointestinal tract but not in the colon, which leads to persistent constipation [124,125]. For instance, in in vitro studies, prolonged exposure to morphine results in downregulation of ß-arrestin2 in the ileum but not in the colon. This implies a role for β-arrestin2 in the development of opioid side effects, which include constipation, as reported in the case of MOR G protein-biased agonists [43,44]. However, morphine tolerance has been measured in β-arrestin2 knockout mouse colon [123], suggesting that the role of β-arrestin2 in the development of opioid tolerance is tissue-dependent, namely it induces tolerance in the CNS and prevents it in the colon. This partially gives an answer to the reduced constipation measured for etorphine and fentanyl in the colon as well as explaining why fentanyl produces less constipation and substantial analgesic tolerance. One possible explanation could be a relatively larger intracellular pool of MOR and, as a consequence, more pronounced receptor recycling. Next, in the colon, morphine triggers dynamin upregulation (i.e., better recycling), yet the activation of the ERK—CREB pathway could also be involved. On the other hand, many splice variants of MOR are described, and alternative splicing of the receptor can influence their ability to activate intracellular pathways. Additionally, the changes to the C-terminal can also influence their internalization and recycling properties. However, the abundance of those splice variants in the colon are not yet well described. Opioid-induced internalization of MORs is related to their ability to induce tolerance after receptor activation. This process involves the phosphorylation of the receptor by different kinases. The kinases involved in this process can vary. Opioid ligands (e.g., fentanyl, endogenous opioids) cause phosphorylation by G-protein receptor kinases (GRKs), but morphine induces phosphorylation by protein kinase C (PKC). After the phosphorylation, the process involves β-arrestin2 binding and internalization occurs. After internalization, the receptor can recycle from the intracellular pool in a few hours. This trafficking is mediated by dynamin. Fast internalization and recycling might provide a protective factor against tolerance development. For further details on opioid tolerance development in the gastrointestinal tract, see the review by Galligan and Sternini [126].

To avoid the consequences of OIC, when drugs such as non-bulk-forming laxatives, prokinetics, and chloride channel activators fail to counteract OIC, drugs that act on opioid receptors as antagonists currently represent the most promising option. Evidence of the involvement of OIC in the development of OID relies on the fact that MOR knocking out or treatment of animals with opioid receptor antagonists with a high affinity for MORs, such as naltrexone-abolished morphine-induced gut dysbiosis in rats, indicates that these effects are dependent on MOR activation [127,128]. According to analysis of the gut microbiome, mice treated with morphine pellets showed an increased proportion of the Firmicutes phylum alongside some bacterial species from this phylum, and this change was counteracted by naltrexone [128]. Likewise, Banerjee and coworkers reported that naltrexone prevents morphine-evoked expansion of Firmicutes, yet naltrexone treatments create a microbial composition similar to control mice but distinct from morphine-treated mice [127]. In another study, naltrexone was also able to reverse the morphine treatment-induced increase in systemic Acinetobacter burdens in mice [129]. Wang’s group also reported that morphine treatment-induced gut dysbiosis, indicated by a reduction in microbial alpha diversity, was normalized by naltrexone [17]. This evidence suggests that long-term MOR activation contributes to microbiome alterations in rodents. In the reviewed studies, naltrexone was applied—from a pharmacokinetic point of view naltrexone is well absorbed in the gastrointestinal tract following oral administration and has reasonable CNS penetration—and would pharmacodynamically counteract the peripheral and the central analgesic effects of opioid analgesics as well as induce withdrawal symptoms in subjects exposed to chronic opioid agonists intended either for therapeutic or illicit use. Moreover, the contribution of the central or peripheral effect of naltrexone to the observed anti-dysbiotic effects is uncertain. To show the involvement of peripheral and central receptors, microbiome studies with opioid analgesics and PAMORAS would be necessary, but as far we know, such results have yet to be published. 

The above-mentioned evidence raises the possible benefit of opioid antagonists that have limited absorption from gastrointestinal tract or have undergone first-pass metabolism once administered orally. These two properties are found in quaternary opioid receptor antagonists and naloxone, respectively. In order to elucidate the contribution of the peripheral MORs to the observed changes in the microbiome, trials with the aforementioned PAMORAs could be of use.

At first glance, the mechanism of OID seems likely due to OIC. Indeed, opioid treatment induces dysbiosis, which in turn contributes to the disruption of the intestinal epithelial barrier and, as result, bacterial translocation from gastrointestinal tract to other organs occurs [21,130,131]. This effect has been attributed to a shift in short-chain fatty acids (SCFAs)—(butyrate)-producing bacteria, such as Faecalibacterium [21]. Constipation evokes reduction in SCFA production. In addition, low abundances of Faecalibacterium, Ruminococcaceae, and Roseburia were detected in the feces of constipated people [132]. Firmicutes species, including Lactobacillaceae, Ruminococcaceae, and Lachnospiraceae largely participate in the production of SCFAs [133,134]. Opioid antagonist therapy, as mentioned above, is based on two strategies, namely applying peripherally acting opioid antagonists such methylnaltrexone and pegylated naloxone or opioid analgesics and naloxone in combination [81,91,109,135,136]. In general, constipation, and particularly chronic constipation, causes alterations in the composition of the gut microbiome that affect the production of SCFAs, which have a principal role in gastrointestinal motility. In addition, endogenous gastrointestinal motility promotors such as motilin and gastrin have been reported to be decreased in the serum of patients with constipation [137,138]. Likewise, treatment with opioid agonists inhibits gastrointestinal motility by direct or indirect action through modulating the release of neurotransmitters involved in gastrointestinal peristalsis [139]. This indicates that MOR antagonists, once applied prior to or simultaneously with opioid analgesics, would inhibit gastrointestinal changes including constipation. Studies have provided evidence for the involvement of intestinal bacteria in the development of neuropathic pain [20,22,118]. However, to the best of our knowledge, the extent of the influence and the type of bacterial phylum involved in the development of different neuropathic pain entities have not been reported in a single paper. Current data regarding the efficacy of opioids in the treatment of neuropathic pain are controversial. On the other hand, opioid analgesics are the mainstay of moderate to severe cancer pain management. In neuropathic animal pain models, several studies have shown that a reduction in MORs which is manifested by a reduction in the efficacy of opioids [12,140,141,142,143]. Indeed, the relationship between neuropathic pain and opioid analgesic-induced gastrointestinal dysbiosis remains unelucidated. Nevertheless, recently, several studies have shown that treatment with opioid analgesics causes alterations in microbiome composition that may provoke the development of the adverse effects of opioids, including opioid analgesic tolerance [4,17,18,23,144]. To achieve adequate opioid analgesia, dose escalation is required, which further aggravates the side effects, including OIC as the main factor altering gut microbiome composition, as reviewed above. In the last four decades, outstanding studies have laid the research foundations for the distribution of functional MORs in the peripheral tissues that mediate antinociceptive effects of systemically or locally administered opioid agonists [3,5,7,25,40,145,146,147]. In these studies, several opioid agonists with limited CNS penetration have been proven to produce peripheral antinociceptives; however, the central side effects (addiction liability, tolerance) were not fully elucidated. Indeed, developing opioid analgesics with limited central side effects is a great clinical need and challenge. The question raised is, how peripherally acting opioid agonists could affect gut microbiome composition. Therefore, the hypothesis is that opioid antagonists whose action is localized to the gastrointestinal tract would be of great interest since they differ from CNS-penetrating antagonists in avoiding inhibition of MORs in the CNS or periphery, particularly those located outside of the intestinal luminal surface to mediate peripheral antinociceptive effects.

Logically, treatment with future peripheral opioid analgesics will be associated with OIC and, as a consequence, changes in microbiome composition might occur. However, to the best of our knowledge, no study has investigated this issue. Loperamide, a peripherally acting opioid agonist, and its antimotility effect via the activation of MORs, has been utilized in the treatment of diarrhea. This characteristic can be used as a tool to predict future scenario-related OIC and dysbiosis. However, as has recently been noted, developing biased opioid analgesics may forego OIC [148]. Indeed, loperamide is often used in preclinical microbiota studies, mostly as a tool to induce constipation and detect constipation-related microbiome changes [149,150,151,152,153,154,155,156,157,158,159,160,161]. Findings from these studies have shown inconsistent results related to microbiome composition due to the use of different doses of loperamide. For instance, the ratio of the two main phyla Firmicutes/Bacteroidetes did not change consistently. Nevertheless, hitherto, the majority of studies have proved a decrease in Bifidobacteria, Lactobacillus, and Ruminococcus, but the level of Bacteroides was found to be either decreased, unchanged, or increased in different publications. In contrast with what has already been mentioned, Proteobacteria was mostly unchanged following loperamide treatment. On the other hand, in treatment with loperamide, similar to centrally acting opioid analgesic treatment, microbiota studies [127,128,162] showed that the integrity of the intestinal barrier was also disrupted in some cases, which can lead to inflammation [153,159,163]. Overall, loperamide-induced intestinal dysbiosis was similar to that induced by classical opioid analgesics (morphine or others). This further supports the recent data from other works that have shown similarities in the intestinal bacterial dysbiosis evoked by opioid analgesics and opioid antidiarrheal agent treatments [23]. In addition, OIC and constipation not related to opioid use cause the intestinal dysbiosis of overlapping (Table 2). Thus, even if a clinically effective peripherally acting opioid analgesic is developed, it will logically cause similar gastrointestinal side effects and microbiome changes as loperamide or a CNS-acting opioid agonist, when not considering the impact of future biased opioid agonists. These data again indicate that the particularly promising possibility is the use of gastrointestinal-tract-restricted opioid antagonists such as oral naloxone. Furthermore, these types of antagonists may counteract the action of other compounds showing an opioid-mediated effect in the context of OID.

With respect to the promising effect of opioid antagonists against human intestinal dysbiosis caused by opioid analgesic treatment, a clinical trial by Gicquelais et al. has investigated this scenario. In this study, 46 outpatients from an addiction treatment facility were enrolled in the investigation. They were subdivided into four groups namely, opioid agonists (such as heroin or prescription opioids), antagonists (such as naltrexone), agonist–antagonist combinations (such as buprenorphine and naloxone), and neither opioid agonists nor antagonists being used during the time of sample collection. Comparing people who used neither agonists nor antagonists, it was found that those who used opioid agonists only had reduced alpha diversity and different bacterial community profiles. Roseburia, unclassified Firmicutes, and Bilophila were less abundant in the agonist group compared to those using neither agonists nor antagonists. In the agonist group, the relative abundances of Clostridium cluster XIVa, unclassified Firmicutes, Lactobacillus, Faecalicoccus, Anaerostipes, and Streptococcus were higher compared to the group using neither agonists nor antagonists. There were no differences in gut microbiota characteristics between people using agonists + antagonists, antagonists only, and neither agonists nor antagonists. These results suggest that partial opioid agonists may have a different effect on the microbiota than full opioid agonists. In addition, the effects of opioids on the gut microbiota may be counteracted by naltrexone or naloxone [164]. Treatment of patients with OID is of urgent medical need and establishing a future therapy scheme including opioid antagonists with restricted gastrointestinal effects would decrease patient compliance, opioid analgesic tolerance, and intestinal dysbiosis-related pain behaviors.

**Table 2:** Changes in the microbiome composition of subjects with OIC or with constipation not related to opioid use.

Bacteria	Opioid Treatment	Reference	Note Subject	Constipation	Reference	Note Type of Constipation
*Firmicutes* *Bacteroidetes*	↑ ↓	[165] [163]	Sprague Dawley rat (oxycodone 2 mg/kg s.c. twice a day for 5 days)	↑ ↓	[166]	Irritable bowel syndrome patients with constipation.
			C57BL/6 mouse (loperamide 9.6 mg/kg p.o., twice a day for 14 days)			
*Bifidobacterium*, *Lactobacillus*	↓	[167]	C57BL/6 mouse (escalating doses of morphine from 5 to 40 mg/kg, twice/day for 8 days)	↓	[168]	Patients with functional constipation
*Lactobacillus*, *Bacteroides* and *Akkermansia*	↓ ↑	[169]	C57BL/6 mouse (hydromorphone 7.5 mg/kg twice a day for 8 days)	↓ ↑	[96]	Mice received fecal microbiota from patients with constipation
*Bacteroidetes*, *Lactobacillus*, *and Clostridium*	↓	[127]	C57BL/6 mouse (25 mg morphine pellet implanted for 3 days)	↓	[168,170]	Adult patients with functional constipation
*Ruminococcus*, *Clostridium* spp.	↑	[171]	C57BL/6 mouse (intermittent and sustained morphine)	↑	[172]	Children with functional constipation
*Roseburia* *Enterobacteriaceae*	↓ ↑	[164]	Patients (heroin or prescription opioids)	↓ ↑	[173]	Patients with constipated-irritable bowel syndrome
*Roseburia*	↓	[164] [174]	Patients (heroin or prescription opioids)	↓	[175] [176] [177]	Patients with severe chronic constipation Italian subjects with functional constipation
			C57BL/6 pregnant mouse (10 mg/kg hydromorphone i.p. for 3 days, on gestation days G11-G13)			Constipated Women of Reproductive Age

## 5. Exploring the Possible Interaction between Food-Derived Opioids or Probiotics and Opioid Analgesics in Microbiome Composition

The question is raised whether food, especially milk and fermented dairy product-derived opioids (e.g., yogurt, kefir) could affect intestinal microbiome composition, particularly during treatment with opioid analgesics. Bovine milk-derived β-casomorphins display agonist activity on MORs [178] measured by in vitro assays, and have shown antinociceptive activity in in vivo assays. Likewise, bovine milk-derived α-casein exorphins have shown opioid agonist activity. In contrast, casoxins derived from both bovine and human κ- and α-caseins have been proven as opioid antagonists. This means that the modulation of the intestinal microbiota composition could also occur alongside the digestion of milk products. Theoretically, peptides with a opioid agonist character could enhance the effects of opioid drugs due to their ability to interact with MORs, leading to the enhancement of both the desired and undesired opioid-related effects. In this regard, the consumption of β-casomorphine-7 and its propeptide has been reported to display gastrointestinal effects manifesting as delays in transit time, cramping, increased mucus production, and increased production of inflammatory mediators [179]. On the other hand, those peptides with an opioid antagonist character could ameliorate the effects of both current opioid analgesics and the above-mentioned food-derived opioids. With respect to the composition of the gut microbiome, some studies have shown that the abundance of beneficial genera Lactobacillus and Bifidobacterium increased in humans taking dairy products (milk, yogurt, and kefir) [180,181,182,183]. On the other hand, the abundance of these bacteria either decreased or increased both in human and animals subjected to opioid treatments [23]. Indeed, the direction of the shift in microbiome composition when opioid analgesics are taken alone or in combination with a peripherally acting opioid antagonist, taken simultaneously with dairy products, to the best of our knowledge, has not been elucidated yet. It is worth noting that enzymatic digestion of milk-derived peptides can also result in the formation of peptides with antibacterial activity [184]. The later data further complicate the scenario when opioid analgesics are administered simultaneously with dairy products.

Probiotics have recently attracted the attention of researchers, since they show positive impacts on OIC, OID, opioid use disorder, and opioid analgesia. Furthermore, they have shown to be beneficial in several functional and neurodegenerative CNS disorders such Alzheimer’s disease, major depressive disorder, epilepsy, Parkinson’s disease, multiple sclerosis, and schizophrenia [185,186] as presented in Table 3.

## 6. Conclusions and Future Perspectives

**Figure 2:**
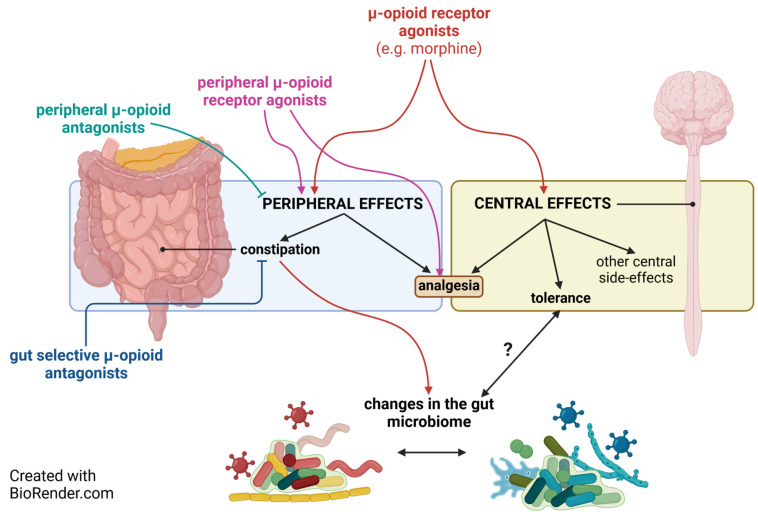
The pharmacology of opioid agonists and gut-selective MOR antagonists in relation to constipation, dysbiosis, and analgesia.

The clinically available MOR analgesics or future opioid agonists that are intended to produce peripheral antinociception once administered orally, OIC and its consequences in relation to dysbiosis, should be accounted for (Figure 2). Therefore, developing opioid antagonists with localized action on gastrointestinal tract (gut-selective µ-opioid antagonists) would be clinically relevant in the context of the drawback of dysbiosis in the development of opioid-related adverse effects. That is, they would reverse the OIC-evoked dysbiosis and meanwhile avoid affecting the central analgesia and tolerance as well as the peripheral opioid analgesia. Current relevant opioid analgesics produce central analgesia, whereas peripherally acting opioid agonists have been proposed as future analgesics. Opioid antagonists with localized action on the gastrointestinal tract, once combined with opioid analgesics and with or without probiotics, may have clinical value in the management of pain.

## Figures and Tables

**Table 3:** Human and animal results on the impact of probiotics on the presented CNS diseases, pain, OUD, and constipation.

Disease	Probiotics	Subject	Outcomes	+/−	Reference
**Alzheimer’s** **disease**	Lactobacillus and Bifidobacterium species	Rats	Restoration of synaptic plasticity in the hippocampus of the brain after 56 days of probiotic supplementation.	+	[187]
		Rats	Enhances brain signal transmission by normalizing long-term potentiation, decreases the activation of microglial markers, and increases the expression of BDNF and synapsin. Improvement in cognitive function and spatial learning.	+	[188]
	Multispecies probiotics containing different stains and species of the genera Lactobacillus and Bifidobacterium.	Human	Patients did not respond positively to a blend of six probiotic strains.	−	[189]
**Major** **depressive** **disorder**	*Lactobacillus plantarum* PS128	Mice	Probiotic reduced depression and anxiety in mice, with increased dopamine and serotonin levels.	+	[190]
	*Akkermansia muciniphila*	Mice	Probiotics alleviated depressive-like symptoms in mice by reversing abnormalities in the gut microbiota.	+	[191]
	*Clostridium butyricum* (CBM588) as an adjunctive therapy to the antidepressant drugs	Human	A significant improvement in depression scores.	+	[192]
	*Bifidobacterium longum* NCC3001	Human	Probiotics reduced depression, although not anxiety, in IBS patients and improved their quality of life.	+	[193]
	*L. helveticus* R0052 and *B. longum* R0175	Human	Probiotics did not alleviate depressive symptoms in individuals with low mood who were not on psychotropic medications.	−	[194]
**Epilepsy**	a mixture of pro/prebiotics and vitamins for one month	Rats	Probiotics did not significantly affect the duration and number of spike-and-wave discharges.	−	[195]
	*Lactobacillus rhamnosus*, *Lactobacillus reuteri*, and *Bifidobacterium infantis* for three weeks	Rats	Probiotics reduced oxidative stress, increased antioxidant capacity in the brain, raised inhibitory GABA levels, and improved spatial learning and memory.	+	[196]
	VSL#3 for a month	Rats	A decrease in the frequency and duration of spike–wave discharges, probiotics exhibited anti-inflammatory properties by reducing the levels of SOX2 and neurotrophic factors while increasing the levels of inflammatory factors, alleviating the concurrent anxious and depressive-like behaviors.	+	[197]
	*B. longum*, *L. acidophilus*, and *E. faecalis*	Human	Probiotics reduced seizures, with no notable differences in cognitive function, including measures of intelligence and memory. Probiotics decrease anxiety and depression while improving the quality of life.	+	[198]
**Parkinson’s** **disease**	6 strains (*Bifidobacterium bifidum*, *Bifidobacterium longum*, *Lactobacillus rhamnosis*, *Lactobacillus rhamnosus* GG, rhamnosus GG, *Lactobacillus plantarum* LP28, and *Lactococcus lactis* subsp. Lactis)	Mice	Probiotics induced better motor performance (gait, balance, and coordination) in animals, from week 16 until the end of the experiment at week 24 and mitigated the degeneration of nigral dopaminergic neurons.	+	[199]
	A fermented milk containing probiotics and prebiotics	Human	Fermented milk containing probiotics and prebiotics significantly enhanced bowel movements in individuals with severe constipation linked to parkinson’s disease.	+	[200]
	*Bifidobacterium bifidum*, *Lactobacillus acidophilus*, *Lactobacillus fermentum*, and *Lactobacillus reuteri* over a 12-week period	Human	Probiotics improved the symptoms of patients with Parkinson’s disease measured using total MDS-UPDRS scores.	+	[201]
**Pain**	Mixed probiotic formulation SLAB51	Mice	Probiotics improved paclitaxel-induced mechanical and cold hypersensitivity and increased the levels of opioid and cannabinoid receptors in the spinal cord.	+	[202]
	14-strainprobiotic mixture for 8 weeks in chronic and 10 weeks in episodic migraines	Human	Improvements in the frequency and severity of migraines and reduction in the consumption of abortive medications despite no significant changes in serum levels of selected inflammatory biomarkers	+	[203]
**Multiple** **sclerosis**	*Lactobacillus plantarum* A7, *Bifidobacterium animalis* PTCC 1631 or a mixture of both strains for 22 days beginning simultaneous with induction EAE	Mice	Probiotics ameliorated experimental autoimmune encephalomyelitis, in an animal model of multiple sclerosis, through inhibiting disease-associated cytokines while increasing anti-inflammatory cytokines.	+	[204]
	VSL3 probiotic mixture, which includes Lactobacillus, Bifidobacterium, and Streptococcus	Human	Probiotics resulted in a shift in their gut microbiota that helps to modulate the anti-inflammatory response of the peripheral innate immune system by regulating the intermediate monocytes.	+	[205]
**Schizophrenia**	*Bifidobacterium longum* for 2 weeks	Mice	Probiotics showed promise in alleviating schizophrenia symptoms by reducing apomorphine-induced rearing behavior, lowering plasma corticosterone levels, and decreasing the kynurenine-to-tryptophan ratio.	+	[206]
	*Bifidobacterium breve* A-1 for four weeks	Human	Probiotics improved positive and negative syndrome scale (PANSS) scores, reduced anxiety and depression, and increased IFN-g, IL-1R1, IL-10, and IL-22 levels while decreasing TNF-a levels.	+	[207]
	Lactobacilli and *Bifidobacterium bifidum* was given with vitamin D	Human	Improvement in the general and total PANSS scores, decreased circulating CRP levels and enhanced total antioxidant capacity of plasma, indicating symptomatic improvement and reduced inflammation.	+	[208]
	*Lactobacillus rhamnosus* and *Bifidobacterium lactis* Bb12 for 14 weeks	Human	Probiotics did not change PANSS scores over the course of the 14-week trial though increased plasma BDNF.	−	[209]
**Opioid** **use** **disorders**	VSL#3	Mice	Morphine-tolerant mice displayed a reduction in Bifidobacteriaceae and Lactobacillaceae at the family level and Bifidobacterium and Lactobacillus at the genus level. The probiotic VSL#3 pre-treatment prevented morphine-induced dysbiosis and so attenuated morphine tolerance in both tail flick and hot plate assays.	+	[18]
	*Bifidobacterium longum* subspecies longum 35624™ or *Lactobacillus rhamnosus* GG	Mice	Probiotic treatment does not alter naloxone-precipitated withdrawal in morphine-dependent mice.	−	[210]
**Constipation**	probiotic chocolate containing *Streptococcus thermophilus* MG510 and *Lactobacillus plantarum* LRCC5193	Rats	Loperamide-induced constipation was associated with a relative increase in the abundance of the family Enterobacteriaceae and a decrease in the genera Bifidobacterium and Lactobacillus, the Clostridium group (cluster IV) as well as *F. prausnitzii*. Probiotic administration could modulate the gut microbiota.	+	[149]
	Multi-strain probiotics containing a mixture of (*Lactobacillus plantarum* PBS067, *Lactobacillus rhamnosus* LRH020, *Bifidobacterium animalis* subsp. lactis BL050; Roelmi HPC), *L. plantarum* UALp05, *Lactobacillus acidophilus* DDS-1, and *Streptococcus thermophilus* CKDB027	Rats	Multi-strain probiotics alleviate loperamide-induced constipation by adjusting the microbiome, serotonin, and short-chain fatty acids in rats. The abundances of the phylum Verrucomicrobia, the family Erysipelotrichaceae, and the genus Akkermansia were increased in fecal samples of the probiotic-treated groups.	+	[211]
	two probiotic cocktails (One formulation with *Lactobacillus acidophilus*, *Bifidobacterium bifidum* and *Lactobacillus rhamnosus*; and another with *Lactobacillus acidophilus*, *Bifidobacterium bifidum*, *Lactobacillus rhamnosus*, *Lactobacillus paracasei*, *Bifidobacterium longum*, *Bifidobacterium lactis*, *Lactobacillus casei*, *Bifidobacterium animallis*)	Human	Functional constipation symptoms improved with the two probiotic cocktails, which increased weekly evacuation and stool quality.	+	[212]

## Data Availability

Not applicable.

## References

[1] Krishnamurti C., Rao S.S.C.C. (2016). The isolation of morphine by serturner. Indian J. Anaesth..

[2] Pasternak G.W. (1998). The central questions in pain perception may be peripheral. Proc. Natl. Acad. Sci. USA.

[3] Balogh M., Zádori Z.S., Lázár B., Karádi D., László S., Mousa S.A., Hosztafi S., Zádor F., Riba P., Schäfer M. (2018). The peripheral versus central antinociception of a novel opioid agonist: Acute inflammatory pain in rats. Neurochem. Res..

[4] Fürst S., Zádori Z.S., Zádor F., Király K., Balogh M., László S.B., Hutka B., Mohammadzadeh A., Calabrese C., Galambos A.R. (2020). On the role of peripheral sensory and gut mu opioid receptors: Peripheral analgesia and tolerance. Molecules.

[5] Khalefa B.I., Mousa S.A., Shaqura M., Lackó E., Hosztafi S., Riba P., Schäfer M., Ferdinandy P., Fürst S., Al-Khrasani M. (2013). Peripheral antinociceptive efficacy and potency of a novel opioid compound 14-O-MeM6SU in comparison to known peptide and non-peptide opioid agonists in a rat model of inflammatory pain. Eur. J. Pharmacol..

[6] Lackó E., Riba P., Giricz Z., Váradi A., Cornic L., Balogh M., Király K., Cseko K., Mousa S.A., Hosztafi S. (2016). New morphine analogs produce peripheral antinociception within a certain dose range of their systemic administration. J. Pharmacol. Exp. Ther..

[7] Schmidhammer H., Al-Khrasani M., Fürst S., Spetea M. (2023). Peripheralization Strategies Applied to Morphinans and Implications for Improved Treatment of Pain. Molecules.

[8] Tegeder I., Meier S., Burian M., Schmidt H., Geisslinger G., Lötsch J., LoÈtsch J. (2003). Peripheral opioid analgesia in experimental human pain models. Brain.

[9] Stein C., Clark J.D., Oh U., Vasko M.R., Wilcox G.L., Overland A.C., Vanderah T.W., Spencer R.H. (2009). Peripheral mechanisms of pain and analgesia. Brain Res. Rev..

[10] Al-Khrasani M., Lackó E., Riba P., Király K., Sobor M., Timár J., Mousa S., Schäfer M., Fürst S. (2012). The central versus peripheral antinociceptive effects of μ-opioid receptor agonists in the new model of rat visceral pain. Brain Res. Bull..

[11] Bauer C.S., Nieto-Rostro M., Rahman W., Tran-Van-Minh A., Ferron L., Douglas L., Kadurin I., Ranjan Y.S., Fernandez-Alacid L., Millar N.S. (2009). The increased trafficking of the calcium channel subunit α2δ-l to presynaptic terminals in neuropathic pain is inhibited by the α2δ ligand pregabalin. J. Neurosci..

[12] Balogh M., Varga B.K., Karádi D.Á., Riba P., Puskár Z., Kozsurek M., Al-Khrasani M., Király K., Martínez-Navarro M. (2019). Maldonado, R.Similarity and dissimilarity in antinociceptive effects of dipeptidyl-peptidase 4 inhibitors, Diprotin A and vildagliptin in rat inflammatory pain models following spinal administration. Eur. J. Pain.

[13] Martínez-Navarro M., Maldonado R., Baños J. (2019). Why mu-opioid agonists have less analgesic efficacy in neuropathic pain?. Eur. J. Pain.

[14] DeHaven-Hudkins D.L., DeHaven R.N., Little P.J., Techner L.M. (2008). The involvement of the μ-opioid receptor in gastrointestinal pathophysiology: Therapeutic opportunities for antagonism at this receptor. Pharmacol. Ther..

[15] Martin W.R.F., Correll C.U., Weiden P.J., Jiang Y., Pathak S., Di Petrillo L., Silverman B.L., Ehrich E.W., Katsiki N., Hatzitolios A.I. (2022). Extraction of neonatal rat myocardium—HHS Public Access. Sci. Rep..

[16] Holzer P. (2009). Opioid receptors in the gastrointestinal tract. Regul. Pept..

[17] Wang F., Meng J., Zhang L., Johnson T., Chen C., Roy S. (2018). Morphine induces changes in the gut microbiome and metabolome in a morphine dependence model. Sci. Rep..

[18] Zhang L., Meng J., Ban Y., Jalodia R., Chupikova I., Fernandez I., Brito N., Sharma U., Abreu M.T., Ramakrishnan S. (2019). Morphine tolerance is attenuated in germfree mice and reversed by probiotics, implicating the role of gut microbiome. Proc. Natl. Acad. Sci. USA.

[19] Kang M., Mischel R.A., Bhave S., Komla E., Cho A., Huang C., Dewey W.L., Akbarali H.I. (2017). The effect of gut microbiome on tolerance to morphine mediated antinociception in mice. Sci. Rep..

[20] Lin B., Wang Y., Zhang P., Yuan Y., Zhang Y., Chen G. (2020). Gut microbiota regulates neuropathic pain: Potential mechanisms and therapeutic strategy. J. Headache Pain.

[21] Thomas K.R., Watt J., Wu C.M.J., Akinrinoye A., Amjad S., Colvin L., Cowe R., Duncan S.H., Russell W.R., Forget P. (2022). Pain and Opioid-Induced Gut Microbial Dysbiosis. Biomedicines.

[22] Pane K., Boccella S., Guida F., Franzese M., Maione S., Salvatore M. (2022). Role of gut microbiota in neuropathy and neuropathic pain states: A systematic preclinical review. Neurobiol. Dis..

[23] Zádori Z.S., Király K., Al-Khrasani M., Gyires K. (2023). Interactions between NSAIDs, opioids and the gut microbiota—Future perspectives in the management of inflammation and pain. Pharmacol. Ther..

[24] Minami M., Satoh M. (1995). Molecular biology of the opioid receptors: Structures, functions and distributions. Neurosci. Res..

[25] Stein C., Schäfer M., Machelska H. (2003). Attacking pain at its source: New perspectives on opioids. Nat. Med..

[26] Minami M. (2004). Molecular pharmacology of opioid receptors. Folia Pharmacol. Jpn..

[27] Leaney J.L., Tinker A. (2000). The role of members of the pertussis toxin-sensitive family of G proteins in coupling receptors to the activation of the G protein-gated inwardly rectifying potassium channel. Proc. Natl. Acad. Sci. USA.

[28] Cox B.M. (2013). Recent developments in the study of opioid receptors. Mol. Pharmacol..

[29] Wei L.-N., Loh H.H. (2002). Regulation of opioid receptor expression. Curr. Opin. Pharmacol..

[30] Henriksen G., Willoch F. (2008). Imaging of opioid receptors in the central nervous system. Brain.

[31] Koneru A., Satyanarayana S., Rizwan S. (2009). Endogenous opioids: Their physiological role and receptors. Glob. J. Pharmacol..

[32] Martyn J.A.J., Mao J., Bittner E.A. (2019). Opioid Tolerance in Critical Illness. Reply. N. Engl. J. Med..

[33] Mori T., Shibasaki Y., Matsumoto K., Shibasaki M., Hasegawa M., Wang E., Masukawa D., Yoshizawa K., Horie S., Suzuki T. (2013). Mechanisms that underlie μ-opioid receptor agonist-induced constipation: Differential involvement of μ-opioid receptor sites and responsible regions. J. Pharmacol. Exp. Ther..

[34] Matsumoto K., Umemoto H., Mori T., Akatsu R., Saito S., Tashima K., Shibasaki M., Kato S., Suzuki T., Horie S. (2016). Differences in the morphine-induced inhibition of small and large intestinal transit: Involvement of central and peripheral μ-opioid receptors in mice. Eur. J. Pharmacol..

[35] Manara L., Bianchetti A. (1985). The Central and Peripheral Influences of Opioids on Gastrointestinal Propulsion. Annu. Rev. Pharmacol. Toxicol..

[36] Leppert W. (2012). The impact of opioid analgesics on the gastrointestinal tract function and the current management possibilities. Contemp. Oncol..

[37] De Luca A., Coupar I.M. (1996). Insights into opioid action in the intestinal tract. Pharmacol. Ther..

[38] Friedman J.D., Dello Buono F.A. (2001). Opioid antagonists in the treatment of opioid-induced constipation and pruritus. Ann. Pharmacother..

[39] Greenwood-Van Meerveld B., Gardner C.J., Little P.J., Hicks G.A., Dehaven-Hudkins D.L. (2004). Preclinical studies of opioids and opioid antagonists on gastrointestinal function. Neurogastroenterol. Motil..

[40] Zádor F., Mohammadzadeh A., Balogh M., Zádori Z.S., Király K., Barsi S., Galambos A.R., László S.B., Hutka B., Váradi A. (2020). Comparisons of in vivo and in vitro opioid effects of newly synthesized 14-methoxycodeine-6-O-sulfate and codeine-6-O-sulfate. Molecules.

[41] Brock C., Olesen S.S., Olesen A.E., Frøkjaer J.B., Andresen T., Drewes A.M. (2012). Opioid-induced bowel dysfunction: Pathophysiology and management. Drugs.

[42] Gillis A., Kliewer A., Kelly E., Henderson G., Christie M.J., Schulz S., Canals M. (2020). Critical Assessment of G Protein-Biased Agonism at the μ-Opioid Receptor. Trends Pharmacol. Sci..

[43] DeWire S.M., Yamashita D.S., Rominger D.H., Liu G., Cowan C.L., Graczyk T.M., Chen X.-T., Pitis P.M., Gotchev D., Yuan C. (2013). A G Protein-Biased Ligand at the μ—Opioid Receptor Is Potently Analgesic with Reduced Gastrointestinal and Respiratory Dysfunction Compared with Morphine. J. Pharmacol. Exp. Ther..

[44] Conibear A.E., Kelly E. (2019). A biased view of μ-Opioid receptors?. Mol. Pharmacol..

[45] Pasternak G.W., Childers S.R., Snyder S.H. (1980). Opiate analgesia: Evidence for mediation by a subpopulation of opiate receptors. Science.

[46] Portoghese P.S. (1965). A New Concept on the Mode of Interaction of Narcotic Analgesics with Receptors. J. Med. Chem..

[47] Wolozin B.L., Pasternak G.W. (1981). Classification of multiple morphine and enkephalin binding sites in the central nervous system. Proc. Natl. Acad. Sci. USA.

[48] Camilleri M. (2011). Opioid-induced constipation: Challenges and therapeutic opportunities. J. Am. Coll. Gastroenterol. ACG.

[49] Kalso E., Edwards J.E., Moore A.R., McQuay H.J. (2004). Opioids in chronic non-cancer pain: Systematic review of efficacy and safety. Pain.

[50] Bell T.J., Panchal S.J., Miaskowski C., Bolge S.C., Milanova T., Williamson R. (2009). The prevalence, severity, and impact of opioid-induced bowel dysfunction: Results of a US and European patient survey (PROBE 1). Pain Med..

[51] Taylor R., Pergolizzi J.V., Porreca F., Raffa R.B. (2013). Opioid antagonists for pain. Expert. Opin. Investig. Drugs.

[52] Choi Y.S., Billings J.A. (2002). Opioid antagonists: A review of their role in palliative care, focusing on use in opioid-related constipation. J. Pain Symptom Manag..

[53] Greig S.L., Keating G.M. (2015). Naltrexone ER/bupropion ER: A review in obesity management. Drugs.

[54] Mouaffak F., Hamzaoui S., Kebir O., Laqueille X. (2020). Kleptomania treated with naltrexone in a patient with intellectual disability. J. Psychiatry Neurosci..

[55] Sudakin D. (2016). Naltrexone: Not just for opioids anymore. J. Med. Toxicol..

[56] Anderson W.S., Sheth R.N., Bencherif B., Frost J.J., Campbell J.N. (2002). Naloxone increases pain induced by topical capsaicin in healthy human volunteers. Pain.

[57] Brown D.R., Goldberg L.I. (1985). The use of quaternary narcotic antagonists in opiate research. Neuropharmacology.

[58] Bates J.J., Foss J.F., Murphy D.B. (2004). Are peripheral opioid antagonists the solution to opioid side effects?. Anesth. Analg..

[59] Bianchetti A., Giudice A., Picerno N., Carminati P. (1982). Pharmacological actions of levallorphan allyl bromide (CM 32191), a new peripheral narcotic antagonist. Life Sci..

[60] Cannom R.R., Mason R.J. (2009). Methylnaltrexone: The answer to opioid-induced constipation?. Expert Opin. Pharmacother..

[61] Dragonetti M., Bianchetti A., Sacilotto R., Giudice A., Ferrarese N., Cattaneo C., Manara L. (1983). Levallorphan methyl iodide (SR 58002), a potent narcotic antagonist with peripheral selectivity superior to that of other quaternary compounds. Life Sci..

[62] Ferreira S.H., Lorenzetti B.B., Rae G.A. (1984). Is methylnalorphinium the prototype of an ideal peripheral analgesic?. Eur. J. Pharmacol..

[63] Manara L., Bianchi G., Fiocchi R., Notarnicola A., Peracchia F., Tavani A. (1982). Inhibition of gastrointestinal transit by morphine and FK 33-824 in the rat and comparative narcotic antagonist properties of naloxone and its N-methyl quaternary analog. Life Sci..

[64] Notarnicola A., Landi M., Bianchi G., Tavani A. (1983). Relative ability of N-methyl nalorphine and N-methyl levallorphan to prevent antinociception and intestinal transit inhibition in morphine treated rats. Life Sci..

[65] Russell J., Bass P., Goldberg L.I., Schuster C.R., Merz H. (1982). Antagonism of gut, but not central effects of morphine with quaternary narcotic antagonists. Eur. J. Pharmacol..

[66] Yuan C.-S., Foss J.F., Moss J. (1995). Effects of methylnaltrexone on morphine-induced inhibition of contraction in isolated guinea-pig ileum and human intestine. Eur. J. Pharmacol..

[67] Tavani A., Bianchi G., Manara L. (1979). Morphine no longer blocks gastrointestinal transit but retains antinociceptive action in diallylnormorphine-pretreated rats. Eur. J. Pharmacol..

[68] Anselmi L., Huynh J., Vegezzi G., Sternini C. (2013). Effects of methylnaltrexone on guinea pig gastrointestinal motility. Naunyn-Schmiedeberg’s Arch. Pharmacol..

[69] Yuan Y., Stevens D.L., Braithwaite A., Scoggins K.L., Bilsky E.J., Akbarali H.I., Dewey W.L., Zhang Y. (2012). 6β-N-heterocyclic substituted naltrexamine derivative NAP as a potential lead to develop peripheral mu opioid receptor selective antagonists. Bioorg. Med. Chem. Lett..

[70] Li G., Aschenbach L.C., Chen J., Cassidy M.P., Stevens D.L., Gabra B.H., Selley D.E., Dewey W.L., Westkaemper R.B., Zhang Y. (2009). Design, synthesis, and biological evaluation of 6alpha- and 6beta-N-heterocyclic substituted naltrexamine derivatives as mu opioid receptor selective antagonists. J. Med. Chem..

[71] Williams D.A., Zheng Y., David B., Yuan Y., Zaidi S.A., Stevens D.L., Scoggins K.L., Selley D.E., Dewey W.L., Akbarali H.I. (2016). 6β-N-Heterocyclic Substituted Naltrexamine Derivative BNAP: A Peripherally Selective Mixed MOR/KOR Ligand. ACS Chem. Neurosci..

[72] Kanemasa T., Koike K., Arai T., Ono H., Horita N., Chiba H., Nakamura A., Morioka Y., Kihara T., Hasegawa M. (2019). Pharmacologic effects of naldemedine, a peripherally acting μ-opioid receptor antagonist, in in vitro and in vivo models of opioid-induced constipation. Neurogastroenterol. Motil..

[73] Floettmann E., Bui K., Sostek M., Payza K., Eldon M. (2017). Pharmacologic profile of naloxegol, a peripherally acting μ-opioid receptor antagonist, for the treatment of opioid-induced constipation. J. Pharmacol. Exp. Ther..

[74] Zimmerman D.M., Gidda J.S., Cantrell B.E., Schoepp D.D., Johnson B.G., Leander J.D. (1994). Discovery of a potent, peripherally selective trans-3, 4-dimethyl-4-(3-hydroxyphenyl) piperidine opioid antagonist for the treatment of gastrointestinal motility disorders. J. Med. Chem..

[75] Zimmerman D.M. (1994). LY246736 Dihydrate. m Opioid receptor antagonist. Drugs Future.

[76] Spetea M., Rief S.B., Ben Haddou T., Fink M., Kristeva E., Mittendorfer H., Haas S., Hummer N., Follia V., Guerrieri E. (2019). Synthesis, Biological, and Structural Explorations of New Zwitterionic Derivatives of 14-O-Methyloxymorphone, as Potent μ/δ Opioid Agonists and Peripherally Selective Antinociceptives. J. Med. Chem..

[77] Zádor F., Balogh M., Váradi A., Zádori Z.S., Király K., Szűcs E., Varga B., Lázár B., Hosztafi S., Riba P. (2017). 14-O-Methylmorphine: A Novel Selective Mu-Opioid Receptor Agonist with High Efficacy and Affinity. Eur. J. Pharmacol..

[78] Yuan C.S., Foss J.F., Osinski J., Toledano A., Roizen M.F., Moss J. (1997). The safety and efficacy of oral methylnaltrexone in preventing morphine-induced delay in oral-cecal transit time. Clin. Pharmacol. Ther..

[79] Yuan C.S., Foss J.F., O’Connor M., Toledano A., Roizen M.F., Moss J. (1996). Methylnaltrexone prevents morphine-induced delay in oral-cecal transit time without affecting analgesia: A double-blind randomized placebo-controlled trial. Clin. Pharmacol. Ther..

[80] Yuan C.S., Foss J.F., O’Connor M., Osinski J., Karrison T., Moss J., Roizen M.F. (2000). Methylnaltrexone for reversal of constipation due to chronic methadone use: A randomized controlled trial. JAMA.

[81] Viscusi E.R. (2019). Clinical Overview and Considerations for the Management of Opioid-induced Constipation in Patients With Chronic Noncancer Pain. Clin. J. Pain.

[82] Gregorian T., Lewis J., Tsu L. (2017). Opioid-Induced Constipation: Clinical Guidance and Approved Therapies.

[83] Diego L., Atayee R., Helmons P., Von Gunten C.F. (2009). Methylnaltrexone: A novel approach for the management of opioid-induced constipation in patients with advanced illness. Expert. Rev. Gastroenterol. Hepatol..

[84] Michna E., Blonsky E.R., Schulman S., Tzanis E., Manley A., Zhang H., Iyer S., Randazzo B. (2011). Subcutaneous methylnaltrexone for treatment of opioid-induced constipation in patients with chronic, nonmalignant pain: A randomized controlled study. J. Pain.

[85] Rauck R., Slatkin N.E., Stambler N., Harper J.R., Israel R.J. (2017). Randomized, Double-Blind Trial of Oral Methylnaltrexone for the Treatment of Opioid-Induced Constipation in Patients with Chronic Noncancer Pain. Pain Pract..

[86] Chey W.D., Webster L., Sostek M., Lappalainen J., Barker P.N., Tack J. (2014). Naloxegol for Opioid-Induced Constipation in Patients with Noncancer Pain. N. Engl. J. Med..

[87] Hale M., Wild J., Reddy J., Yamada T., Arjona Ferreira J.C. (2017). Naldemedine versus placebo for opioid-induced constipation (COMPOSE-1 and COMPOSE-2): Two multicentre, phase 3, double-blind, randomised, parallel-group trials. Lancet Gastroenterol. Hepatol..

[88] Camilleri M., Hale M., Morlion B., Tack J., Webster L., Wild J. (2021). Naldemedine improves patient-reported outcomes of opioid-induced constipation in patients with chronic non-cancer pain in the compose phase 3 studies. J. Pain Res..

[89] Katakami N., Harada T., Murata T., Shinozaki K., Tsutsumi M., Yokota T., Narabayashi M., Boku N. (2017). Randomized phase III and extension studies of naldemedine in patients with opioid-induced constipation and cancer. J. Clin. Oncol..

[90] Löwenstein O., Leyendecker P., Lux E.A., Blagden M., Simpson K.H., Hopp M., Bosse B., Reimer K. (2010). Efficacy and safety of combined prolonged-release oxycodone and naloxone in the management of moderate/severe chronic non-malignant pain: Results of a prospectively designed pooled analysis of two randomised, double-blind clinical trials. BMC Clin. Pharmacol..

[91] Meissner W., Leyendecker P., Mueller-Lissner S., Nadstawek J., Hopp M., Ruckes C., Wirz S., Fleischer W., Reimer K. (2009). A randomised controlled trial with prolonged-release oral oxycodone and naloxone to prevent and reverse opioid-induced constipation. Eur. J. Pain.

[92] Callaghan J.T., Cerimele B., Nowak T.V., DeLong A., Myhart E., Oldham S. (1998). Effect of the opioid antagonist ly 246736 on gastro-intestinal transit in human subjects. Gastroenterology.

[93] Liu S.S., Hodgson P.S., Carpenter R.L., Fricke J.R. (2001). ADL 8-2698, a trans-3, 4-dimethyl-4-(3-hydroxyphenyl) piperidine, prevents gastrointestinal effects of intravenous morphine without affecting analgesia. Clin. Pharmacol. Ther..

[94] Webster L., Jansen J.P., Peppin J., Lasko B., Irving G., Morlion B., Snidow J., Pierce A., Mortensen E., Kleoudis C. (2008). Alvimopan, a peripherally acting mu-opioid receptor (PAM-OR) antagonist for the treatment of opioid-induced bowel dysfunction: Results from a randomized, double-blind, placebo-controlled, dose-finding study in subjects taking opioids for chronic non-cance. PAIN®.

[95] Jansen J.P., Lorch D., Langan J., Lasko B., Hermanns K., Kleoudis C.S., Snidow J.W., Pierce A., Wurzelmann J., Mortensen E.R. (2011). A randomized, placebo-controlled phase 3 trial (study sb-767905/012) of alvimopan for opioid-induced bowel dysfunction in patients with non-cancer pain. J. Pain.

[96] Cao H., Liu X., An Y., Zhou G., Liu Y., Xu M., Dong W., Wang S., Yan F., Jiang K. (2017). Dysbiosis contributes to chronic constipation development via regulation of serotonin transporter in the intestine. Sci. Rep..

[97] Webster L.R., Michna E., Khan A., Israel R.J., Harper J.R. (2017). Long-Term Safety and Efficacy of Subcutaneous Methylnaltrexone in Patients with Opioid-Induced Constipation and Chronic Noncancer Pain: A Phase 3, Open-Label Trial. Pain. Med..

[98] Rauck R.L., Slatkin N.E., Stambler N., Israel R.J. (2018). Safety of oral methylnaltrexone for opioid-induced constipation in patients with chronic noncancer pain. J. Pain Res..

[99] Webster L., Chey W.D., Tack J., Lappalainen J., Diva U., Sostek M. (2014). Randomised clinical trial: The long-term safety and tolerability of naloxegol in patients with pain and opioid-induced constipation. Aliment. Pharmacol. Ther..

[100] Webster L., Dhar S., Eldon M., Masuoka L., Lappalainen J., Sostek M. (2013). A phase 2, double-blind, randomized, placebo-controlled, dose-escalation study to evaluate the efficacy, safety, and tolerability of naloxegol in patients with opioid-induced constipation. Pain.

[101] Webster L.R., Nalamachu S., Morlion B., Reddy J., Baba Y., Yamada T., Ferreira J.C.A. (2018). Long-term use of naldemedine in the treatment of opioid-induced constipation in patients with chronic noncancer pain: A randomized, double-blind, placebo-controlled phase 3 study. Pain.

[102] Guerriero F., Roberto A., Greco M.T., Sgarlata C., Rollone M., Corli O. (2016). Long-term efficacy and safety of oxycodone–naloxone prolonged release in geriatric patients with moderate-to-severe chronic noncancer pain: A 52-week open-label extension phase study. Drug Des. Devel. Ther..

[103] Siemens W., Gaertner J., Becker G. (2015). Advances in pharmacotherapy for opioid-induced constipation–a systematic review. Expert. Opin. Pharmacother..

[104] Brenner D.M., Chey W.D. (2014). An evidence-based review of novel and emerging therapies for constipation in patients taking opioid analgesics. Am. J. Gastroenterol. Suppl..

[105] Bui K., Zhou D., Sostek M., She F., Al-Huniti N. (2016). Effects of CYP3A modulators on the pharmacokinetics of naloxegol. J. Clin. Pharmacol..

[106] Bui K., She F., Sostek M. (2014). The effects of renal impairment on the pharmacokinetics, safety, and tolerability of naloxegol. J. Clin. Pharmacol..

[107] Chandrasekaran A., Tong Z., Li H., Erve J.C.L., DeMaio W., Goljer I., McConnell O., Rotshteyn Y., Hultin T., Talaat R. (2010). Metabolism of intravenous methylnaltrexone in mice, rats, dogs, and humans. Drug Metab. Dispos..

[108] Rotshteyn Y., Boyd T.A., Yuan C.-S. (2011). Methylnaltrexone bromide: Research update of pharmacokinetics following parenteral administration. Expert. Opin. Drug Metab. Toxicol..

[109] Mueller-Lissner S. (2010). Fixed combination of oxycodone with naloxone: A new way to prevent and treat opioid-induced constipation. Adv. Ther..

[110] Thomas J. (2008). Opioid-Induced Bowel Dysfunction. J. Pain Symptom Manag..

[111] Neefjes E.C.W., Van Der Wijngaart H., Van Der Vorst M.J.D.L., Ten Oever D., Van Der Vliet H.J., Beeker A., Rhodius C.A., Van Den Berg H.P., Berkhof J., Verheul H.M.W. (2019). Optimal treatment of opioid induced constipation in daily clinical practice—An observational study. BMC Palliat. Care..

[112] Chamberlain B.H., Rhiner M., Slatkin N.E., Stambler N., Israel R.J. (2021). Subcutaneous methylnaltrexone for treatment of opioid-induced constipation in cancer versus noncancer patients: An analysis of efficacy and safety variables from two studies. J. Pain Res..

[113] Mori M., Ji Y., Kumar S., Ashikaga T., Ades S. (2017). Phase II trial of subcutaneous methylnaltrexone in the treatment of severe opioid-induced constipation (OIC) in cancer patients: An exploratory study. Int. J. Clin. Oncol..

[114] Nalamachu S.R., Pergolizzi J., Taylor R., Slatkin N.E., Barrett A.C., Yu J., Bortey E., Paterson C., Forbes W.P. (2015). Efficacy and Tolerability of Subcutaneous Methylnaltrexone in Patients with Advanced Illness and Opioid-Induced Constipation: A Responder Analysis of 2 Randomized, Placebo-Controlled Trials. Pain Pract..

[115] Mo J., Michl P., Witt H., Scholz M., Fe C., Biology C., Medicine N., Munich H.C. (2017). UEG Week 2017 Oral Presentations. United Eur. Gastroenterol. J..

[116] Carding S., Verbeke K., Vipond D.T., Corfe B.M., Owen L.J. (2015). Dysbiosis of the gut microbiota in disease. Microb. Ecol. Health Dis..

[117] Luca M., Chattipakorn S.C., Sriwichaiin S., Luca A. (2020). Cognitive-behavioural correlates of dysbiosis: A review. Int. J. Mol. Sci..

[118] Guo R., Chen L.H., Xing C., Liu T. (2019). Pain regulation by gut microbiota: Molecular mechanisms and therapeutic potential. Br. J. Anaesth..

[119] Dworsky-Fried Z., Kerr B.J., Taylor A.M.W. (2020). Microbes, microglia, and pain. Neurobiol. Pain.

[120] Ustianowska K., Ustianowski Ł., Machaj F., Gorący A., Rosik J., Szostak B., Szostak J., Pawlik A. (2022). The Role of the Human Microbiome in the Pathogenesis of Pain. Int. J. Mol. Sci..

[121] Moriarty O., Ruane N., O’Gorman D., Maharaj C.H., Mitchell C., Sarma K.M., Finn D.P., McGuire B.E. (2017). Cognitive Impairment in Patients with Chronic Neuropathic or Radicular Pain: An Interaction of Pain and Age. Front. Behav. Neurosci..

[122] Khera T., Rangasamy V. (2021). Cognition and Pain: A Review. Front. Psychol..

[123] Akbarali H.I., Inkisar A., Dewey W.L. (2014). Site and mechanism of morphine tolerance in the gastrointestinal tract. Neurogastroenterol. Motil..

[124] Nelson A.D., Camilleri M. (2016). Opioid-induced constipation: Advances and clinical guidance. Ther. Adv. Chronic. Dis..

[125] Ross G.R., Gabra B.H., Dewey W.L., Akbarali H.I. (2008). Morphine tolerance in the mouse ileum and colon. J. Pharmacol. Exp. Ther..

[126] Galligan J.J., Sternini C. (2017). Insights into the role of opioid receptors in the GI tract: Experimental evidence and therapeutic relevance. Gastrointest. Pharmacol..

[127] Banerjee S., Sindberg G., Wang F., Meng J., Sharma U., Zhang L., Dauer P., Chen C., Dalluge J., Johnson T. (2016). Opioid-induced gut microbial disruption and bile dysregulation leads to gut barrier compromise and sustained systemic inflammation. Mucosal. Immunol..

[128] Meng J., Banerjee S., Li D., Sindberg G.M., Wang F., Ma J., Roy S. (2015). Opioid exacerbation of gram-positive sepsis, induced by gut microbial modulation, is rescued by IL-17A neutralization. Sci. Rep..

[129] Breslow J.M., Monroy M.A., Daly J.M., Meissler J.J., Gaughan J., Adler M.W., Eisenstein T.K. (2011). Morphine, but not trauma, sensitizes to systemic acinetobacter baumannii infection. J. Neuroimmune Pharmacol..

[130] Meng J., Sindberg G.M., Roy S. (2015). Disruption of Gut Homeostasis by Opioids Accelerates HIV Disease Progression. Front. Microbiol..

[131] Meng J., Yu H., Ma J., Wang J., Banerjee S., Charboneau R., Barke R.A., Roy S. (2013). Morphine induces bacterial translocation in mice by compromising intestinal barrier function in a TLR-dependent manner. PLoS ONE.

[132] Zhuang M., Shang W., Ma Q., Strappe P., Zhou Z. (2019). Abundance of Probiotics and Butyrate-Production Microbiome Manages Constipation via Short-Chain Fatty Acids Production and Hormones Secretion. Mol. Nutr. Food Res..

[133] Fusco W., Lorenzo M.B., Cintoni M., Porcari S., Rinninella E., Kaitsas F., Lener E., Mele M.C., Gasbarrini A., Collado M.C. (2023). Short-Chain Fatty-Acid-Producing Bacteria: Key Components of the Human Gut Microbiota. Nutrients.

[134] Biddle A., Stewart L., Blanchard J., Leschine S. (2013). Untangling the Genetic Basis of Fibrolytic Specialization by Lachnospiraceae and Ruminococcaceae in Diverse Gut Communities. Diversity.

[135] Lang-Illievich K., Bornemann-Cimenti H. (2019). Opioid-induced constipation: A narrative review of therapeutic options in clinical management. Korean J. Pain.

[136] Morlion B.J., Mueller-Lissner S.A., Vellucci R., Leppert W., Coffin B.C., Dickerson S.L., O’Brien T. (2018). Oral Prolonged-Release Oxycodone/Naloxone for Managing Pain and Opioid-Induced Constipation: A Review of the Evidence. Pain Pract..

[137] Kim J.E., Kang M.J., Choi J.Y., Park J.J., Lee M.R., Song B.R., Kim H.R., Park J.W., Choi H.J., Bae S.J. (2018). Regulation of gastrointestinal hormones during laxative activity of gallotannin-enriched extract isolated from Galla Rhois in loperamide-induced constipation of SD rats. Lab. Anim. Res..

[138] Preston D.M., Adrian T.E., Christofides N.D., Lennard-Jones J.E., Bloom S.R. (1985). Positive correlation between symptoms and circulating motilin, pancreatic polypeptide and gastrin concentrations in functional bowel disorders. Gut.

[139] Davis M.P. (2005). The opioid bowel syndrome: A review of pathophysiology and treatment. J. Opioid Manag..

[140] Jones A.K.P., Watabe H., Cunningham V.J., Jones T. (2004). Cerebral decreases in opioid receptor binding in patients with central neuropathic pain measured by [11C] diprenorphine binding and PET. Eur. J. Pain.

[141] Maarrawi J., Peyron R., Mertens P., Costes N., Magnin M., Sindou M., Laurent B., Garcia-Larrea L. (2007). Differential brain opioid receptor availability in central and peripheral neuropathic pain. Pain.

[142] Obara I., Parkitna J.R., Korostynski M., Makuch W., Kaminska D., Przewlocka B., Przewlocki R. (2009). Local peripheral opioid effects and expression of opioid genes in the spinal cord and dorsal root ganglia in neuropathic and inflammatory pain. PAIN®.

[143] Shaqura M., Khalefa B.I., Shakibaei M., Winkler J., Al-Khrasani M., Fürst S., Mousa S.A., Schäfer M. (2013). Reduced number, G protein coupling, and antinociceptive efficacy of spinal mu-opioid receptors in diabetic rats are reversed by nerve growth factor. J. Pain.

[144] Akbarali H.I., Dewey W.L. (2019). Gastrointestinal motility, dysbiosis and opioid-induced tolerance: Is there a link?. Nat. Rev. Gastroenterol. Hepatol..

[145] Al-Khrasani M., Spetea M., Friedmann T., Riba P., Király K., Schmidhammer H., Furst S. (2007). DAMGO and 6β-glycine substituted 14-O-methyloxymorphone but not morphine show peripheral, preemptive antinociception after systemic administration in a mouse visceral pain model and high intrinsic efficacy in the isolated rat vas deferens. Brain Res. Bull..

[146] Fürst S., Riba P., Friedmann T., Tímar J., Al-Khrasani M., Obara I., Makuch W., Spetea M., Schütz J., Przewlocki R. (2005). Peripheral versus Central Antinociceptive Actions of 6-Amino Acid-Substituted Derivatives of 14-O-Methyloxymorphone in Acute and Inflammatory Pain in the Rat. J. Pharmacol. Exp. Ther..

[147] Khalefa B.I., Shaqura M., Al-Khrasani M., Fürst S., Mousa S.A., Schäfer M. (2012). Relative contributions of peripheral versus supraspinal or spinal opioid receptors to the antinociception of systemic opioids. Eur. J. Pain.

[148] Azevedo Neto J., Costanzini A., De Giorgio R., Lambert D.G., Ruzza C., Calò G. (2020). Biased versus Partial Agonism in the Search for Safer Opioid Analgesics. Molecules.

[149] Eor J.Y., Tan P.L., Lim S.M., Choi D.H., Yoon S.M., Yang S.Y., Kim S.H. (2019). Laxative effect of probiotic chocolate on loperamide-induced constipation in rats. Food Res. Int..

[150] Kim M.G., Jo K., Chang Y.B., Suh H.J., Hong K.B. (2020). Changes in the gut microbiome after galactooligosaccharide administration in loperamideinduced constipation. J. Pers. Med..

[151] Inatomi T., Honma M. (2021). Effects of probiotics on loperamide-induced constipation in rats. Sci. Rep..

[152] Hao M., Song J., Zhai X., Cheng N., Xu C., Gui S., Chen J. (2023). Improvement of loperamide-hydrochloride-induced intestinal motility disturbance by Platycodon grandiflorum polysaccharides through effects on gut microbes and colonic serotonin. Front. Cell Infect. Microbiol..

[153] Kim J.-E., Choi Y.-J., Lee S.-J., Gong J.-E., Jin Y.-J., Park S.-H., Lee H.-S., Choi Y.-W., Hong J.-T., Hwang D.-Y. (2021). Laxative Effects of Phlorotannins Derived from Ecklonia cava on Loperamide-Induced Constipation in SD Rats. Molecules.

[154] Kashyap P.C., Marcobal A., Ursell L.K., Larauche M., Duboc H., Earle K.A., Sonnenburg E.D., Ferreyra J.A., Higginbottom S.K., Million M. (2013). Complex Interactions Among Diet, Gastrointestinal Transit, and Gut Microbiota in Humanized Mice. Gastroenterology.

[155] Touw K., Ringus D.L., Hubert N., Wang Y., Leone V.A., Nadimpalli A., Theriault B.R., Huang Y.E., Tune J.D., Herring P.B. (2017). Mutual reinforcement of pathophysiological host-microbe interactions in intestinal stasis models. Physiol. Rep..

[156] Hwang N., Eom T., Gupta S.K., Jeong S.-Y., Jeong D.-Y., Kim Y.S., Lee J.-H., Sadowsky M.J., Tatsuya Unno T. (2017). Genes and Gut Bacteria Involved in Luminal Butyrate Reduction Caused by Diet and Loperamide. Genes.

[157] Deng Y., Li M., Mei L., Cong L.M., Liu Y., Zhang B.B., He C.Y., Zheng P.Y., Yuan J.L. (2018). Manipulation of intestinal dysbiosis by a bacterial mixture ameliorates loperamide-induced constipation in rats. Benef. Microbes..

[158] Li Y., Long S., Liu Q., Ma H., Li J., Xiaoqing W., Yuan J., Li M., Hou B. (2020). Gut microbiota is involved in the alleviation of loperamide-induced constipation by honey supplementation in mice. Food Sci. Nutr..

[159] Kim M.G., Jo K., Cho K., Park S.S., Suh H.J., Hong K.-B. (2021). Prebiotics/Probiotics Mixture Induced Changes in Cecal Microbiome and Intestinal Morphology Alleviated the Loperamide-Induced Constipation in Rat. Food Sci. Anim. Resour..

[160] Liang Y., Wang Y., Wen P., Chen Y., Ouyang D., Wang D., Zhang B., Deng J., Chen Y., Sun Y. (2022). The Anti-Constipation Effects of Raffino-Oligosaccharide on Gut Function in Mice Using Neurotransmitter Analyses, 16S rRNA Sequencing and Targeted Screening. Molecules.

[161] Makizaki Y., Uemoto T., Yokota H., Yamamoto M., Tanaka Y., Ohno H. (2021). Improvement of loperamide-induced slow transit constipation by Bifidobacterium bifidum G9-1 is mediated by the correction of butyrate production and neurotransmitter profile due to improvement in dysbiosis. PLoS ONE.

[162] Wang F., Meng J., Zhang L., Roy S. (2020). Opioid use potentiates the virulence of hospital-acquired infection, increases systemic bacterial dissemination and exacerbates gut dysbiosis in a murine model of Citrobacter rodentium infection. Gut Microbes.

[163] Lin X., Liu Y., Ma L., Ma X., Shen L., Ma X., Chen Z., Chen H., Li D., Su Z. (2021). Constipation induced gut microbiota dysbiosis exacerbates experimental autoimmune encephalomyelitis in C57BL/6 mice. J. Transl. Med..

[164] Gicquelais R.E., Bohnert A.S.B., Thomas L., Foxman B. (2020). Opioid agonist and antagonist use and the gut microbiota: Associations among people in addiction treatment. Sci. Rep..

[165] Simpson S., Kimbrough A., Boomhower B., McLellan R., Hughes M., Shankar K., de Guglielmo G., George O. (2020). Depletion of the Microbiome Alters the Recruitment of Neuronal Ensembles of Oxycodone Intoxication and Withdrawal. eNeuro.

[166] Rajilić-Stojanović M., Biagi E., Heilig H.G.H.J., Kajander K., Kekkonen R.A., Tims S., de Vos W.M. (2011). Global and deep molecular analysis of microbiota signatures in fecal samples from patients with irritable bowel syndrome. Gastroenterology.

[167] Zhang L., Han R., Zhang X., Fang G., Chen J., Li J., Xu S., Qian L., Chen W., Pan F. (2019). Fecal microbiota in patients with ankylosing spondylitis: Correlation with dietary factors and disease activity. Clin. Chim. Acta.

[168] Khalif I.L., Quigley E.M.M.M., Konovitch E.A., Maximova I.D. (2005). Alterations in the colonic flora and intestinal permeability and evidence of immune activation in chronic constipation. Dig. Liver Dis..

[169] Sharma U., Olson R.K., Erhart F.N., Zhang L., Meng J., Segura B., Banerjee S., Sharma M., Saluja A.K., Ramakrishnan S. (2020). Prescription Opioids induce Gut Dysbiosis and Exacerbate Colitis in a Murine Model of Inflammatory Bowel Disease. J. Crohns Colitis..

[170] Kim S.-E., Choi S.C., Park K.S., Park M.I., Shin J.E., Lee T.H., Jung K.W., Koo H.S., Myung S.-J. (2015). Change of Fecal Flora and Effectiveness of the Short-term VSL#3 Probiotic Treatment in Patients With Functional Constipation. J. Neurogastroenterol. Motil..

[171] Lee K., Vuong H.E., Nusbaum D.J., Hsiao E.Y., Evans C.J., Taylor A.M.W. (2018). The gut microbiota mediates reward and sensory responses associated with regimen-selective morphine dependence. Neuropsychopharmacology.

[172] Zhu L., Liu W., Alkhouri R., Baker R.D., Bard J.E., Quigley E.M., Baker S.S. (2014). Structural changes in the gut microbiome of constipated patients. Physiol. Genom..

[173] Chassard C., Dapoigny M., Scott K.P., Crouzet L., Del’homme C., Marquet P., Martin J.C., Pickering G., Ardid D., Eschalier A. (2012). Functional dysbiosis within the gut microbiota of patients with constipated-irritable bowel syndrome. Aliment. Pharmacol. Ther..

[174] Abu Y., Tao J., Dutta R., Yan Y., Vitari N., Kolli U., Roy S. (2022). Brief Hydromorphone Exposure During Pregnancy Sufficient to Induce Maternal and Neonatal Microbial Dysbiosis. J. Neuroimmune Pharmacol..

[175] Yarullina D.R., Shafigullin M.U., Sakulin K.A., Arzamastseva A.A., Shaidullov I.F., Markelova M.I., Grigoryeva T.V., Karpukhin O.Y., Sitdikova G.F. (2020). Characterization of gut contractility and microbiota in patients with severe chronic constipation. PLoS ONE.

[176] Mancabelli L., Milani C., Lugli G.A., Turroni F., Mangifesta M., Viappiani A., Ticinesi A., Nouvenne A., Meschi T., van Sinderen D. (2017). Unveiling the gut microbiota composition and functionality associated with constipation through metagenomic analyses. Sci. Rep..

[177] Li H., Chen J., Ren X., Yang C., Liu S., Bai X., Shan S., Dong X. (2021). Gut Microbiota Composition Changes in Constipated Women of Reproductive Age. Front. Cell Infect. Microbiol..

[178] Brantl V., Teschemacher H., Bläsig J., Henschen A., Lottspeich F. (1981). Opioid activities of β-casomorphins. Life Sci..

[179] De Vasconcelos M.L., Oliveira L.M.F.S., Hill J.P., Vidal A.M.C. (2023). Difficulties in Establishing the Adverse Effects of β-Casomorphin-7 Released from β-Casein Variants—A Review. Foods.

[180] Odamaki T., Sugahara H., Yonezawa S., Yaeshima T., Iwatsuki K., Tanabe S., Tominaga T., Togashi H., Benno Y., Xiao J. (2012). Effect of the oral intake of yogurt containing Bifidobacterium longum BB536 on the cell numbers of enterotoxigenic Bacteroides fragilis in microbiota. Anaerobe.

[181] Odamaki T., Kato K., Sugahara H., Xiao J.Z., Abe F., Benno Y. (2016). Effect of probiotic yoghurt on animal-based diet-induced change in gut microbiota: An open, randomised, parallel-group study. Benef. Microbes..

[182] Link-Amster H., Rochat F., Saudan K.Y., Mignot O., Aeschlimann J.M. (1994). Modulation of a specific humoral immune response and changes in intestinal flora mediated through fermented milk intake. FEMS Immunol. Med. Microbiol..

[183] Yılmaz İ., Dolar M.E., Özpınar H. (2019). Effect of administering kefir on the changes in fecal microbiota and symptoms of inflammatory bowel disease: A randomized controlled trial. Turk. J. Gastroenterol..

[184] Nagpal R., Behare P., Rana R., Kumar A., Kumar M., Arora S., Morotta F., Jain S., Yadav H. (2011). Bioactive peptides derived from milk proteins and their health beneficial potentials: An update. Food Funct..

[185] Ojha S., Patil N., Jain M., Kole C., Kaushik P. (2023). Probiotics for Neurodegenerative Diseases: A Systemic Review. Microorganisms.

[186] Thangaleela S., Sivamaruthi B.S., Kesika P., Chaiyasut C. (2022). Role of Probiotics and Diet in the Management of Neurological Diseases and Mood States: A Review. Microorganisms.

[187] Rezaei Asl Z., Sepehri G., Salami M. (2019). Probiotic treatment improves the impaired spatial cognitive performance and restores synaptic plasticity in an animal model of Alzheimer’s disease. Behav. Brain Res..

[188] O’Hagan C., Li J.V., Marchesi J.R., Plummer S., Garaiova I., Good M.A. (2017). Long-term multi-species Lactobacillus and Bifidobacterium dietary supplement enhances memory and changes regional brain metabolites in middle-aged rats. Neurobiol. Learn Mem..

[189] Agahi A., Hamidi G.A., Daneshvar R., Hamdieh M., Soheili M., Alinaghipour A., Esmaeili Taba S.M., Salami M. (2018). Does Severity of Alzheimer’s Disease Contribute to Its Responsiveness to Modifying Gut Microbiota? A Double Blind Clinical Trial. Front. Neurol..

[190] Liu Y.-W., Liu W.-H., Wu C.-C., Juan Y.-C., Wu Y.-C., Tsai H.-P., Wang S., Tsai Y.-C. (2016). Psychotropic effects of Lactobacillus plantarum PS128 in early life-stressed and naïve adult mice. Brain Res..

[191] Ding Y., Bu F., Chen T., Shi G., Yuan X., Feng Z., Duan Z., Wang R., Zhang S., Wang Q. (2021). A next-generation probiotic: Akkermansia muciniphila ameliorates chronic stress-induced depressive-like behavior in mice by regulating gut microbiota and metabolites. Appl. Microbiol. Biotechnol..

[192] Miyaoka T., Kanayama M., Wake R., Hashioka S., Hayashida M., Nagahama M., Okazaki S., Yamashita S., Miura S., Miki H. (2018). Clostridium butyricum MIYAIRI 588 as Adjunctive Therapy for Treatment-Resistant Major Depressive Disorder: A Prospective Open-Label Trial. Clin. Neuropharmacol..

[193] Pinto-Sanchez M.I., Hall G.B., Ghajar K., Nardelli A., Bolino C., Lau J.T., Martin F.-P., Cominetti O., Welsh C., Rieder A. (2017). Probiotic Bifidobacterium longum NCC3001 Reduces Depression Scores and Alters Brain Activity: A Pilot Study in Patients with Irritable Bowel Syndrome. Gastroenterology.

[194] Romijn A.R., Rucklidge J.J., Kuijer R.G., Frampton C. (2017). A double-blind, randomized, placebo-controlled trial of Lactobacillus helveticus and Bifidobacterium longum for the symptoms of depression. Aust. N. Z. J. Psychiatry.

[195] Akkol S., Doğan M.C., Esenkar D., Doğan H., Karamahmutoğlu T., Onat F. (2017). Effects of Probiotic Consumption on Absence Seizures. Epilepsi J. Turk. Epilepsi Soc..

[196] Bagheri S., Heydari A., Alinaghipour A., Salami M. (2019). Effect of probiotic supplementation on seizure activity and cognitive performance in PTZ-induced chemical kindling. Epilepsy Behav..

[197] Aygun H., Akin A.T., Kızılaslan N., Sumbul O., Karabulut D. (2022). Probiotic supplementation alleviates absence seizures and anxiety-and depression-like behavior in WAG/Rij rat by increasing neurotrophic factors and decreasing proinflammatory cytokines. Epilepsy Behav..

[198] Wang X., Ma R., Liu X., Zhang Y. (2022). Effects of long-term supplementation of probiotics on cognitive function and emotion in temporal lobe epilepsy. Front. Neurol..

[199] Hsieh T.-H., Kuo C.-W., Hsieh K.-H., Shieh M.-J., Peng C.-W., Chen Y.-C., Chang Y.-L., Huang Y.-Z., Chen C.-C., Chang P.-K. (2020). Probiotics alleviate the progressive deterioration of motor functions in a mouse model of Parkinson’s disease. Brain Sci..

[200] Barichella M., Pacchetti C., Bolliri C., Cassani E., Iorio L., Pusani C., Pinelli G., Privitera G., Cesari I., Faierman S.A. (2016). Probiotics prebiotic fiber for constipation associated with Parkinson disease: An, RCT. Neurology.

[201] Tamtaji O.R., Taghizadeh M., Kakhaki R.D., Kouchaki E., Bahmani F., Borzabadi S., Oryan S., Mafi A., Asemi Z. (2019). Clinical and metabolic response to probiotic administration in people with Parkinson’s disease: A randomized, double-blind, placebo-controlled trial. Clin. Nutr..

[202] Cuozzo M., Castelli V., Avagliano C., Cimini A., d’Angelo M., Cristiano C., Russo R. (2021). Effects of chronic oral probiotic treatment in paclitaxel-induced neuropathic pain. Biomedicines.

[203] Martami F., Togha M., Seifishahpar M., Ghorbani Z., Ansari H., Karimi T., Jahromi S.R. (2019). The effects of a multispecies probiotic supplement on inflammatory markers and episodic and chronic migraine characteristics: A randomized double-blind controlled trial. Cephalalgia.

[204] Salehipour Z., Haghmorad D., Sankian M., Rastin M., Nosratabadi R., Dallal M.M.S., Tabasi N., Khazaee M., Nasiraii L.R., Mahmoudi M. (2017). Bifidobacterium animalis in combination with human origin of Lactobacillus plantarum ameliorate neuroinflammation in experimental model of multiple sclerosis by altering CD4+ T cell subset balance. Biomed. Pharmacother..

[205] Tankou S.K., Regev K., Healy B.C., Cox L.M., Tjon E., Kivisakk P., Vanande I.P., Cook S., Gandhi R., Glanz B. (2018). Investigation of probiotics in multiple sclerosis. Mult. Scler. J..

[206] Orikasa S., Nabeshima K., Iwabuchi N., Xiao J.-Z. (2016). Effect of repeated oral administration of Bifidobacterium longum BB536 on apomorphine-induced rearing behavior in mice. Biosci. Microbiota. Food Health.

[207] Okubo R., Koga M., Katsumata N., Odamaki T., Matsuyama S., Oka M., Narita H., Hashimoto N., Kusumi I., Xiao J. (2019). Effect of bifidobacterium breve A-1 on anxiety and depressive symptoms in schizophrenia: A proof-of-concept study. J. Affect. Disord..

[208] Ghaderi A., Banafshe H.R., Mirhosseini N., Moradi M., Karimi M.-A., Mehrzad F., Bahmani F., Asemi Z. (2019). Clinical and metabolic response to vitamin D plus probiotic in schizophrenia patients. BMC Psychiatry.

[209] Dickerson F.B., Stallings C., Origoni A., Katsafanas E., Savage C.L.G., Schweinfurth L.A.B., Goga J., Khushalani S., Yolken R.H. (2014). Effect of probiotic supplementation on schizophrenia symptoms and association with gastrointestinal functioning: A randomized, placebo-controlled trial. Prim. Care Companion CNS Disord..

[210] Thomaz A.C., Iyer V., Woodward T.J., Hohmann A.G. (2021). Fecal microbiota transplantation and antibiotic treatment attenuate naloxone-precipitated opioid withdrawal in morphine-dependent mice. Exp. Neurol..

[211] Jeong J.-J., Ganesan R., Jin Y.-J., Park H.J., Min B.H., Jeong M.K., Yoon S.J., Choi M.R., Choi J., Moon J.H. (2023). Multi-strain probiotics alleviate loperamide-induced constipation by adjusting the microbiome, serotonin, and short-chain fatty acids in rats. Front. Microbiol..

[212] Mitelmão F.C.R., Häckel K., de Cássia Bergamaschi C., Gerenutti M., Silva M.T., Balcão V.M., Vila M.M.D.C. (2022). The effect of probiotics on functional constipation in adults: A randomized, double-blind controlled trial. Medicine.

